# Counter changes with changelessness: cope with SARS-CoV-2 immune evasion by targeting cryptic epitopes

**DOI:** 10.1093/lifemedi/lnac006

**Published:** 2022-06-24

**Authors:** Cheng Li, Tianlei Ying, Dimiter S Dimitrov, Yanling Wu

**Affiliations:** MOE/NHC/CAMS Key Laboratory of Medical Molecular Virology, Shanghai Institute of Infectious Disease and Biosecurity, Shanghai Engineering Research Center for Synthetic Immunology, School of Basic Medical Sciences, Fudan University, Shanghai 200032, China; MOE/NHC/CAMS Key Laboratory of Medical Molecular Virology, Shanghai Institute of Infectious Disease and Biosecurity, Shanghai Engineering Research Center for Synthetic Immunology, School of Basic Medical Sciences, Fudan University, Shanghai 200032, China; Center for Antibody Therapeutics, Division of Infectious Diseases, Department of Medicine, University of Pittsburgh School of Medicine, Pittsburgh, PA 15213-2546, USA; MOE/NHC/CAMS Key Laboratory of Medical Molecular Virology, Shanghai Institute of Infectious Disease and Biosecurity, Shanghai Engineering Research Center for Synthetic Immunology, School of Basic Medical Sciences, Fudan University, Shanghai 200032, China

Antibodies and vaccines are effective weapons in the combat against infectious disease outbreaks. However, viruses that exhibit high variability are easy to gain resistance to immune attack after the accumulation of mutations. Currently, significantly decreased neutralizing potency to new SARS-CoV-2 variants, especially Omicron and its subvariant BA.2, was widely observed in antibody-based therapeutics and serum from vaccinated individuals. Efforts are being made to achieve the long-term goal of universal vaccines and antibodies.

The development of viral neutralizing antibodies was mainly focused on the receptor-binding site (RBS) to block the recognition of host cell. Recently, antibody epitopes buried inside the trimeric interface of influenza virus hemagglutinin (HA), which are distinct from RBS, were revealed by the recognition by an antibody against avian influenza H7N9 virus [1]. This antibody, referred to as m826, displayed robust protection against viral challenge in mice by mediating potent antibody-dependent cell-mediated cytotoxicity (ADCC) effect. After that, various antibodies recognizing epitopes at the HA interface were identified with broad cross-group reactivity, indicating the highly conservative nature of this site [2, 3]. These characteristics of the cryptic site brought the possibility of universal influenza drug and vaccine design. Notably, such epitopes could also be observed in the head trimer interface of SARS-CoV-2 spike protein. The receptor-binding domain (RBD) of SARS-CoV-2 sits on the top of the trimeric spike protein with dynamic orientations rotating between “up” and “down.” The “up” RBD exposing the apex receptor-binding motif (RBM) was responsible for angiotensin-converting enzyme 2 (ACE2) recognition and consequently shedding of S1 subunit, thus facilitating further membrane fusion. Three “up” RBDs from a trimeric spike hold like three petals of a flower, forming a deep steric crevice hidden inside, which means the antibodies need to conquer the steric hindrance among adjacent protomers to get access to these cryptic epitopes. When RBD lies down, its interface will be sheltered, making it totally latent in the surface between RBD and the S2 subunit underneath. Therefore, the cryptic epitopes are only accessible on “up” RBD and are less disclosed to immune system. Taken together, these studies revealed the conformational flexibility and a cryptic epitope buried inside the trimer interface of viral membrane proteins.

**Figure 1.**
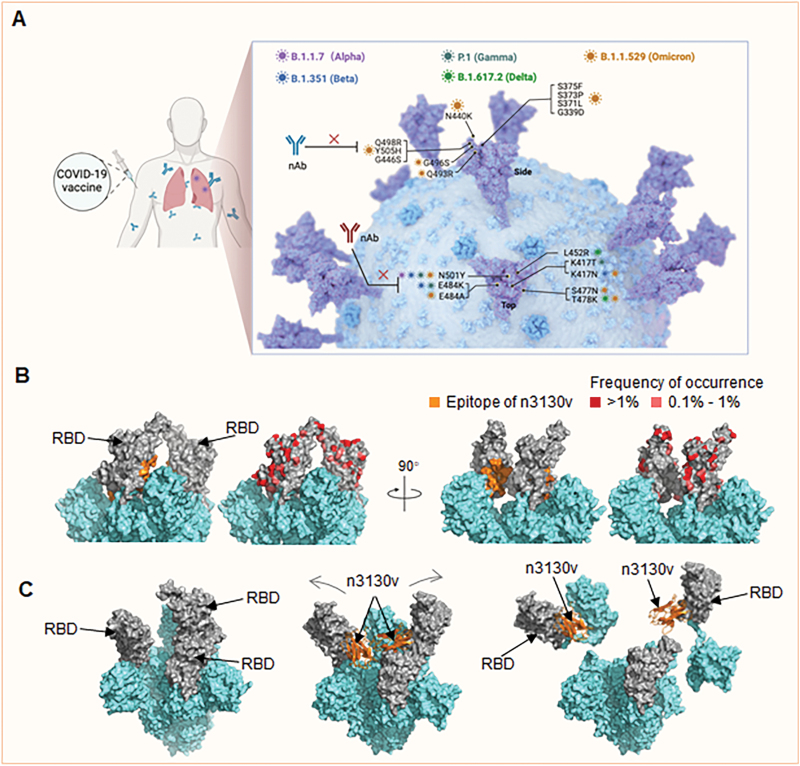
Representative of immune escape of SARS-CoV-2 variants (A), and the highly conserved cryptic antibody epitope buried inside the SARS-CoV-2 spike trimer interface (B, C). Neutralizing potency of serum from vaccinated individuals was reduced due to immune escape of SARSCoV-2 variants, especially the Omicron variant, conferred by RBD mutations (A). The cryptic epitope buried inside spike trimeric interface is highly conserved in SARS-CoV-2 RBD variants (B). The SARS-CoV-2 spike with two RBD in up conformation was shown as surface (PDB 7EB4), epitope of n3130v and mutations in RBDs of frequency >0.1% from public database (https://ngdc.cncb.ac.cn/ncov/variation/spike) were highlighted. The binding of neutralizing antibodies to the cryptic epitope would disrupt spike trimer integrity (C). The SARS-CoV-2 spike with two RBD in up conformation (left). The binding of antibody n3130v led to the outward rotation of “up” RBD towards unstable “wide-up” states (middle) (PDB 7WHK) and the shedding of S1 on viral spike (right).

RNA viruses are known for high frequency of mutations compared to DNA viruses. A tremendous number of SARS-CoV-2 variants have emerged throughout the COVID-19 pandemic, including five most prevalent variants of concern (VOCs), named Alpha (B.1.1.7), Beta (B.1.351), Gamma (P.1), Delta (B.1.617.2), and Omicron (B.1.1.529). Even though countless neutralizing antibodies were generated to fight against viral infections, a recent study showed that Omicron has rendered the majority of neutralizing antibodies ineffective [4], especially antibodies that have epitopes overlapped with the RBM [5]. Analysis of natural mutations on RBD suggested that receptor-binding sites were mutational “hot-spots” with more accumulation of mutations (Fig. 1A). Indeed, RBD is a major target of potent neutralizing antibody responses elicited by vaccination or infection, which may account for the high evolution pressure on this region. Therefore, development of broad-spectrum antibodies to overcome the continuous SARS-CoV-2 antigenic shift conferred by RBD mutations is of urgent need.

Interestingly, the firstly reported SARS-CoV and SARS-CoV-2 cross-reactive antibody CR3022 was found to target a conserved cryptic epitope on RBD of these two viruses [6]. However, although CR3022 showed potent binding ability to SARS-CoV-2 RBD, it negligibly neutralizes SARS-CoV-2 due to the steric hindrance from the adjacent “down” conformation of RBD, N-terminal domain (NTD), or S2 domain underneath RBD in the trimeric spike [7].

Recently, a neutralizing single-domain antibody targeting the cryptic epitopes of SARS-CoV-2 was reported in a study of inhalable bispecific single-domain antibody bn03 [5]. This antibody, designated as n3130v, was generated from a fully human single-domain phage library, and showed competition with CR3022 in binding to RBD. Unlike CR3022, n3130v possessed broadly and potently neutralizing breath across all VOCs. Interestingly, n3130v was able to bind to “one RBD up” spike, which was not accessible for CR3022 because of the clash between the adjacent “down” RBD and CR3022 variable region. Conversely, up-RBD-bound n3130v was even able to interact with the adjacent “down” RBD and thus preventing the formation of three-up state essential for the following membrane fusion. In the bispecific antibody bn03, the interaction between n3130v and “down” RBD pull the “down” RBD inward and synergistically make space for another arm (n3113v) of bn03 to bind on the side surface facing the adjacent NTD. Furthermore, two n3130v antibodies can bind to the same trimeric spike and this binding could disrupt trimer integrity, according to the cryo-electron microscopy structure, by inducing the outward rotation of “up” RBD towards unstable “wide-up” states. These findings explain the neutralizing mechanism of n3130v and other antibodies recognizing this epitope. It is worth noting that despite having overlapped epitopes with CR3022, the smaller size and different binding orientation of the single-domain n3130v may contribute to successfully accessing the trimer interface.

The in-depth investigation of all natural mutations on RBD with occurrence frequency over 0.1% showed that none of them overlap with the epitope of n3130v, further confirming the highly conservative nature of the cryptic n3130v epitope (Fig. 1B). Compared to the immune-dominant RBM, the cryptic epitope is more likely to be masked by the trimeric conformation to protect the vulnerability of the viruses. This leads to less immune attack on the cryptic epitope, and hence rarely occurrence of antigenic drift.

Such cryptic epitopes targeted by n3130v were also identified by another monoclonal antibody S2H97 [8]. Notably, S2H97 exhibits extensively broad binding to RBDs of SARS-CoV, SARS-CoV-2, and even other related ACE2-binding sarbecoviruses. Both of n3130v and S2H97 bind with the loop region of RBD (Phe515-His519 and Pro426-Thr430). Besides, a patch centered on Glu465 in RBD was found mutationally constrained to maintain folded RBD expression and correct quaternary packing with NTD based on previous deep mutational scans [9]. This patch is also in the surface of their binding epitopes, supplementing the conservativeness of the cryptic epitopes.

Altogether, these studies demonstrated the unique superiority of cryptic epitopes as targets of broad-spectrum SARS-CoV-2 antibodies. Until now, most potent neutralizing antibodies are directed against SARS-CoV-2 RBD, which is also immunodominant, raising concerns of immune escape. Particularly, the RBM is known for its variability to avoid most invasion pressure from immune response, according to the evolution course in SARS-CoV and SARS-CoV-2 variants. Identifying a conserved epitope in SARS-CoV-2 variants or even across sarbecoviruses is appealing. Here, we highlight the importance of the recent findings on the highly conserved cryptic epitope concealed in the core of SARS-CoV-2 RBD, as identified by the potent broadly neutralizing antibodies n3130v and S2H97. Antibodies targeting this epitope cannot block ACE2 binding but neutralize through disruption of trimer stability, for which they need to overcome the steric hindrance to get into the right position (Fig. 1C). It is uncommon to separate antibodies targeting this cryptic epitope from immunized people, indicating that it may not be under the selective forces that could lead to antigenic shift. In addition, constrained surface for RBD folding was identified within this epitope. Similar conserved epitopes can also be found in the trimeric interface of influenza virus HA, suggesting that such cryptic epitopes may exist in many viruses beyond SARS-CoV-2 and influenza virus, and serve as ideal targets to counter changing viruses with changelessness broadly neutralizing antibodies and universal vaccines.
